# Pretic-I was a safe and effective artificial cervical disc prosthesis--a retrospective and comparative study with 5-year follow-up

**DOI:** 10.1186/s12891-021-04813-5

**Published:** 2021-11-24

**Authors:** Yingjun Guo, Hao Liu, Jianzhong Xu, Yuxiao Deng, Xiaoliang Tao, Yang Meng, Xiaofei Wang, Ying Hong, Beiyu Wang, Chen Ding, Wenjie Wu

**Affiliations:** 1 Department of Orthopaedics, West China Hospital, Sichuan University, Chengdu, China; 2 Department of Orthopaedics, Southwest Hospital, Third Military Medical University, Chongqing, China

**Keywords:** Cervical disc arthroplasty, Pretic-I, Discover, Adjacent segment disease, Heterotopic ossification

## Abstract

**Background:**

The newly designed cervical disc prosthesis, Pretic-I, had been finished its limited clinical use for over 5 years. At a short-term follow-up of 2 years, we obtained satisfactory clinical results. The long-term clinical efficacy and safety of Pretic-I will now be analyzed.

**Methods:**

Peri-operative parameters included intra-operative blood loss, operation time, off-bed time. Clinical parameters included visual analogue scale (VAS) for arm and neck, neck disability index (NDI), and Japanese Orthopaedic Association (JOA) score. Radiological parameters included C2–7 Cobb angle, Shell angle, and the range of motion (ROM) of C2–7, functional segment unit (FSU), and adjacent FSU. The CDA-related complications included adjacent segment degeneration (ASDeg), adjacent segment disease (ASDis), heterotopic ossification (HO), prosthesis subsidence, prosthesis displacement, and dysphagia.

**Results:**

A total 64 patients from two independent centers received a single-level CDA with Discover (*n* = 32) and Pretic-I (*n* = 32), and all of patients finished a 5-year follow-up. There’re no significant differences between two groups in peri-operative parameters. The clinical parameters improved greatly in Pretic-I group (*p*<0.0001), and there’s no statistical difference from Discover group. Furthermore, Pretic-I could slightly improve the cervical curvature (15.08 ± 11.75 to 18.00 ± 10.61, *p* = 0.3079) and perfectly maintain the Shell angle (3.03 ± 3.68 to 2.23 ± 4.10, *p* = 0.1988), cervical ROM (52.48 ± 14.31 to 53.30 ± 11.71, *p* = 0.8062) and FSU ROM (12.20 ± 4.52 to 10.73 ± 4.45, *p* = 0.2002). The incidence of high-grade HO (Grade III-IV) at the final follow-up was significantly lower in Pretic-I group than in Discover group (12.50% vs. 34.38%, *p* = 0.0389, Statistical Power = 95.36%). The incidences of other CDA-related complications in Pretic-I group were also well-accepted, comparable to the Discover group, without significant differences.

**Conclusion:**

CDA with Pretic-I demonstrated a well-accepted and sustained clinical outcome, with a significantly lower incidence of high-grade HO. This newly designed prosthesis is expected to become an alternative choice for cervical disc prosthesis in the future.

**Supplementary Information:**

The online version contains supplementary material available at 10.1186/s12891-021-04813-5.

## Introduction

Cervical disc degenerative disease (CDDD) is a vital factor affecting the quality of life for the middle-aged and old people [1, 2]. As an important alternative surgical method to treat CDDD, cervical disc arthroplasty (CDA) has been used in clinical practice for decades since 2002 [3, 4], whose clinical effect is similar to that of anterior cervical discectomy and fusion (ACDF). Furthermore, it can reduce the incidence of adjacent segment degeneration (ASDeg) or adjacent segment disease (ASDis) by preserving the range of motion (ROM) of surgical segment to a certain extent [5]. It is now highly recommended in some appropriate circumstances.

With the clinical application of artificial disc prosthesis becoming more and more widespread, several problems of CDA are gradually emerging, such as heterotopic ossification (HO), prosthesis displacement and even falling off, and some scholars also believe that the application of artificial disc prosthesis cannot effectively reduce the incidences of ASDeg and ASDis. Therefore, the assessment of one artificial disc prosthesis will always focus on the incidence of post-operative HO, prosthesis displacement, ASDeg and ASDis, etc. [5–8]. With the deepening of the clinical application and research of artificial disc prosthesis, it was found that many artificial disc prosthesis footprints often do not match the cervical endplates, which may lead to subsidence, displacement, HO and some other complications mentioned above [9].

**Fig. 1:**
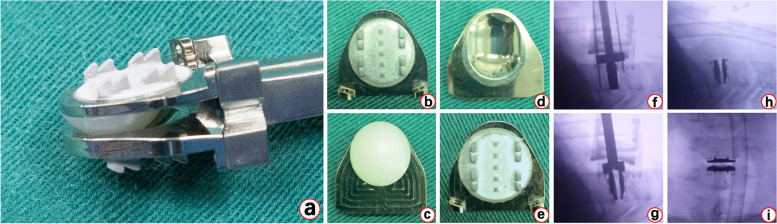
The Images of Pretic-I Cervical Disc Prosthesis and Intra-operative Implantation. **a** Overall design of Pretic-I; **b** and **c** superior and inferior image of the cranial end of Pretic-I; **d** and **e** superior and inferior image of the caudal end of Pretic-I; **f**-**i** Intra-operative use of Pretic-I

In order to better match the prosthesis with the endplate of patients, the new cervical disc prosthesis--Pretic-I was designed and developed as shown in Fig. 1. The previous 2-year follow-up study has shown a positive result, demonstrating its clinical safety and efficacy [10]. The purpose of this study was to verify the long-term effectiveness of Pretic-I. Through a comparative study of 5-year clinical follow-up with Discover prothesis, the clinical efficacy, radiological features and the incidence of CDA-related complications of Pretic-I were analyzed, so as to preliminarily evaluate whether it can meet the standards of large-scale clinical application.

## Methods

### Ethics approval

This study has institutional review board (IRB) approval / research ethics committee approval, and the document has been attached to the submission.

### Study design

By collecting information on a total of 64 patients who underwent single level CDA surgery in two centers from June 2014 to January 2016, a retrospective, comparative, and double-center study was performed. During the follow-up period, the results at 5 time points were examined and evaluated: pre-operative stage, 1 week, 3 months, 1 year and the final follow-up. The inclusion and exclusion criteria were clearly defined (Table 1), which was developed jointly by the surgical teams at both centers. Among the 64 patients, 32 of them who have received Discover prothesis over the same period were enrolled into the control group; while the other 32 of them who received Pretic-I prothesis were enrolled into the experimental group.

**Table 1:** The inclusion and exclusion criteria

Inclusion Criteria	Exclusion Criteria
(1) The patient is over 18 years old; (2) The radiological results and clinical symptoms were consistent with the diagnosis of single-segment disc degeneration; (3) Soft disc herniation; (4) The degeneration located in C3-C7;	(1) There’re spinal protopathy, such as tumor, deformity, and infection; (2) Serious osteoporosis; (3) Rheumatoid arthritis; (4) Ankylosing Spondylitis; (5) Adjacent segment degeneration; (6) Severe narrowing of the degenerative space; (7) Ossification of posterior longitudinal ligaments; (8) The patient’s mental state was unstable and he could not cooperate with the follow-up;

### Surgical technique

Fifty patients (25 patients with Discover and 25 patients with Pretic-I) in our center and 14 patients (7 patients with Discover and 7 patients with Pretic-I) in another center accepted CDA surgery. Prior to the Pretic-I implant, the surgeons at both centers discussed the surgical technique and finalized the procedure and related details. The patient was supine under general anesthesia, and the C-arm was used to determine the position of cervical vertebra and the operative segment. The surgery field was fully exposed by the standard Smith-Robinson approach. After cutting and opening the anterior intervertebral disc annulus fibrosus, nucleus pulposus tissues were removed with nucleus pulposus forceps, and the cartilage endplate were scraped carefully. The dural membrane and bilateral nerve roots were confirmed to be free from compression. X-ray examination results in Fig. 1 showed that the size of the prosthesis was determined with a trial mold, and then the prosthesis of the corresponding size was implanted.

### Clinical evaluation

In peri-operative parameters, intra-operative blood loss and operation time were mainly used to evaluate the operation difficulty of the two prostheses and the damage to local tissues, while off-bed time (the time from operation to their off-bed activity), off-hospital time (the time from operation to their hospital discharge), and back-to-society time (the time from operation to their return to social life) were used to assess the recovery condition after surgery. The clinical effect evaluation mainly included: visual analog score (VAS) for neck and arm, neck disability index (NDI) and Japanese Orthopaedic Association Score (JOA) for cervical spine (only for patients with myelopathy or mixed-type CDDD). In addition, the clinical symptoms associated with ASDis were examined and evaluated to determine the presence of CDDD symptoms of adjacent segments. Dysphagia was also evaluated at every follow-up point. All scores were checked and confirmed by 2 independent and experienced spine surgeons from both centers. After an agreement was reached between the two observers, the data would be finally included.

Furthermore, the recovery rate of each clinical parameters were calculated as follows:

$$\begin{array}{c}\text{VAS}/\text{NDI}\;\text{Recovery}\;\text{Rate}\;=\;\left(\text{pre-operative}\;\text{result}\;-\;\text{final}\;\text{follow-up}\;\text{result}\right)\;/\;\text{pre-operative}\;\text{result}\\\text{JOA}\;\text{Recovery}\;\text{Rate}=\left(\text{final}\;\text{follow-up}\;\text{result}\;-\;\text{pre-operative}\;\text{result}\right)\;/\;\left(17\;-\;\text{pre-operative}\;\text{result}\right)\;\end{array}$$

### Radiological evaluation

At each follow-up point, X-ray and computed tomography (CT) examinations were conducted in neutral and hyperflexion/hyperextension positions. Static parameters included C2–7 Cobb Angle and prosthesis Shell Angle. Dynamic parameters mainly included C2–7 ROM, functional segment unit (FSU) ROM, and upper/lower adjacent FSU ROM (flexion, extension and total ROM).

The presence of ASDeg was assessed based on the modified Hili-brand criteria [11–13] on radiography and CT results, mainly including five diagnostic criteria: (1) intervertebral space narrowing>25%; (2) new or enlarged osteophytes; (3) new disc herniation; (4) endplate sclerosis; and (5) calcification of the anterior/posterior longitudinal ligaments.

In addition to ASDeg, the complications such as HO, prosthesis subsidence and prosthesis displacement were evaluated. The extent of HOs was graded according to the modified McAfee grading system for disc prosthesis as previously described by Mehren et al. [14] (Table 2). Prosthesis subsidence was defined as loss of more than 3 mm on the height of surgical FSU. Prosthesis displacement was defined as horizontal movement with more than 3 mm.

**Table 2:** Modified McAfee’s grading system for heterotopic ossification

Grades	Descriptions
**Grade 0**	No HO present
**Grade I**	HO detectable in front of the vertebral body, but not in the anatomic interdiscal space
**Grade II**	HO extending into the disc space; possible affection of the function of the prosthesis
**Grade III**	Bridging ossifications that still allow movement of the prosthesis
**Grade IV**	Complete fusion of the treated segment without movement in flexion/extension

*HO* Heterotopic ossification

All radiographs images were transferred to a computer as DICOM data, and measurements were performed by 2 independent observers from both centers. After an agreement was reached between the two observers, each parameter was independently measured twice by 2 spine surgeons.

### Statistical analysis

Analysis was conducted using Stata version 13.1 (Stata-Corp LP, College Station, TX). The level of significance was set at p<0.05. All of the clinical and radiological measurements were carried out by two independent and experienced observers. Chi-squared analysis and unpaired t test were used, respectively, for categorical and continuous data between groups, while paired t test was used to compare the data of the one group from different time points. Statistical power with a significant level (alpha) of 0.05 was calculated using G-Power software (version 3.1.9.4) when there was a statistical difference, and 80% is the minimum level for statistical significance [15]. All the data were expressed as mean ± standard deviations or percentages.

## Results

### Patients’ characteristics

A total of 64 patients met the inclusion criteria who agreed to accept single level CDA. The specific data in Table 3 showed no significant difference between the two groups in the basic characteristics. By the end of the final follow-up, no patient needed the second operation for CDDD.

**Table 3:** Patients’ characteristics

	Discover	Pretic-I	Total	***P*** Value
**Number of Cases**	32	32	64	(−)
**Sex (M/F)**	15:17	13:19	28:36	0.6143
**Age (Years)**	42.78 ± 7.38	40.43 ± 6.18	42.36 ± 7.23	0.3023
**Height (cm)**	163.88 ± 7.52	162.91 ± 9.82	163.39 ± 8.76	0.6643
**Weight (kg)**	65.84 ± 10.48	65.29 ± 8.28	65.74 ± 10.12	0.8561
**Smoking History (Y/N)**	16/16	14/18	30/34	0.6164
**Drinking History (Y/N)**	19/13	13/19	32/32	0.1336
**Segment**				0.6884
C3/4	1	1	2	
C4/5	6	3	9	
C5/6	17	17	34	
C6/7	8	11	19	
**Classification of CDDD**				0.3672
Myelopathy	12	15	27	
Radiculopathy	11	6	17	
Mixed-type	9	11	20	
**Follow-up Period (Months)**	57.22 ± 4.76	58.28 ± 5.57	57.75 ± 5.21	0.4225

*CDDD* Cervical disc degenerative disease, *M* Male, *F* Female, *Y* Yes, *N* No

### Peri-operative conditions

In order to evaluate and compare the conditions of the two groups during and after surgery, a statistical analysis of peri-operative parameters was conducted. As shown in Table 4, intra-operative blood loss and operative time in two groups were similar, without statistical differences. In addition, the post-operative recovery between both two groups was compared and analyzed, and as shown in Table 4, there was no significant difference in all of the parameters between the two groups.

**Table 4:** The results of perioperative parameters

	Discover (***n*** = 32)	Pretic-I (***n*** = 32)	***P*** Value
**Intrao-perative Blood Loss (mm)**	28.59 ± 16.07	25.16 ± 13.89	0.3710
**Operation Time (min)**	116.09 ± 9.25	115.16 ± 8.05	0.6718
**Off-bed Time (day)**	1.28 ± 0.45	1.19 ± 0.46	0.4220
**Off-hospital Time (day)**	7.53 ± 1.17	7.38 ± 1.22	0.6087
**Back-to-society Time (day)**	20.94 ± 5.07	20.63 ± 5.83	0.8225

### Clinical results

As shown in Table 5, the evaluation results of various clinical items such as VAS-neck, VAS-arm, NDI, and JOA in the two groups at the final follow-up were significantly better than those before surgery in both groups. The comparison of clinical results between the two groups is shown in Table 6. It was noticed that there was no significant difference with valid statistical power in the results between two groups.

**Table 5:** Clinical recovery of the final follow-up

Items	Groups	Pre-operative	Final Follow-up	Recovery Rate	***P*** Value	Statistical Power
**VAS-neck**	Discover (*n* = 32)	3.91 ± 1.94	0.22 ± 0.41	94.22 ± 12.05%	<0.0001	100.00%
	Pretic-I (*n* = 32)	3.88 ± 1.83	0.19 ± 0.39	96.53 ± 7.56%	<0.0001	100.00%
**VAS-arm**	Discover (*n* = 32)	5.78 ± 1.83	0.41 ± 0.55	92.02 ± 11.98%	<0.0001	100.00%
	Pretic-I (*n* = 32)	5.59 ± 2.26	0.38 ± 0.48	92.83 ± 11.10%	<0.0001	100.00%
**NDI**	Discover (*n* = 32)	24.56 ± 9.53	5.63 ± 2.46	76.07 ± 8.48%	<0.0001	100.00%
	Pretic-I (*n* = 32)	23.16 ± 8.96	5.38 ± 2.38	75.97 ± 8.74%	<0.0001	100.00%
**JOA**	Discover (*n* = 21)	8.14 ± 1.61	16.29 ± 0.76	91.24 ± 10.17%	<0.0001	100.00%
	Pretic-I (*n* = 26)	8.58 ± 1.71	16.04 ± 0.81	87.50 ± 13.32%	<0.0001	100.00%

*VAS* Visual analog score, *NDI* Neck disability index, *JOA* Japanese Orthopaedic Association score

**Table 6:** Follow-up results of clinical parameters

Items	Groups	Pre-operative		3 Months		12 Months		Final Follow-up	
**VAS-neck**	Discover (*n* = 32)	3.91 ± 1.94	*p* = 0.9483	0.75 ± 0.66	*p* = 0.7325	0.38 ± 0.54	*p* = 0.6477	0.22 ± 0.41	*p* = 0.7606
	Pretic-I (*n* = 32)	3.88 ± 1.83		0.69 ± 0.77		0.31 ± 0.53		0.19 ± 0.39	
**VAS-arm**	Discover (*n* = 32)	5.78 ± 1.83	*p* = 0.7211	0.72 ± 0.76	*p* = 0.7565	0.66 ± 0.73	*p* = 0.7402	0.41 ± 0.55	*p* = 0.8133
	Pretic-I (*n* = 32)	5.59 ± 2.26		0.78 ± 0.82		0.59 ± 0.74		0.38 ± 0.48	
**NDI**	Discover (*n* = 32)	24.56 ± 9.53	*p* = 0.5516	11.09 ± 5.44	*p* = 0.7609	8.06 ± 3.58	*p* = 0.3127	5.63 ± 2.46	*p* = 0.6857
	Pretic-I (*n* = 32)	23.16 ± 8.96		10.72 ± 4.13		7.13 ± 3.67		5.38 ± 2.38	
**JOA**	Discover (*n* = 21)	8.14 ± 1.61	*p* = 0.3903	13.90 ± 1.11	***p*** = 0.0327* **SP = 54.73%**	15.14 ± 0.71	*p* = 0.8445	16.29 ± 0.76	*p* = 0.3015
	Pretic-I (*n* = 26)	8.58 ± 1.71		13.04 ± 1.45		15.08 ± 1.36		16.04 ± 0.81	

*VAS* Visual analog score, *NDI* Neck disability index, *JOA* Japanese Orthopaedic Association score, *SP* Statistical power

* indicates that the data has a *P* value less than 0.05

### Radiological results

**Fig. 2:**
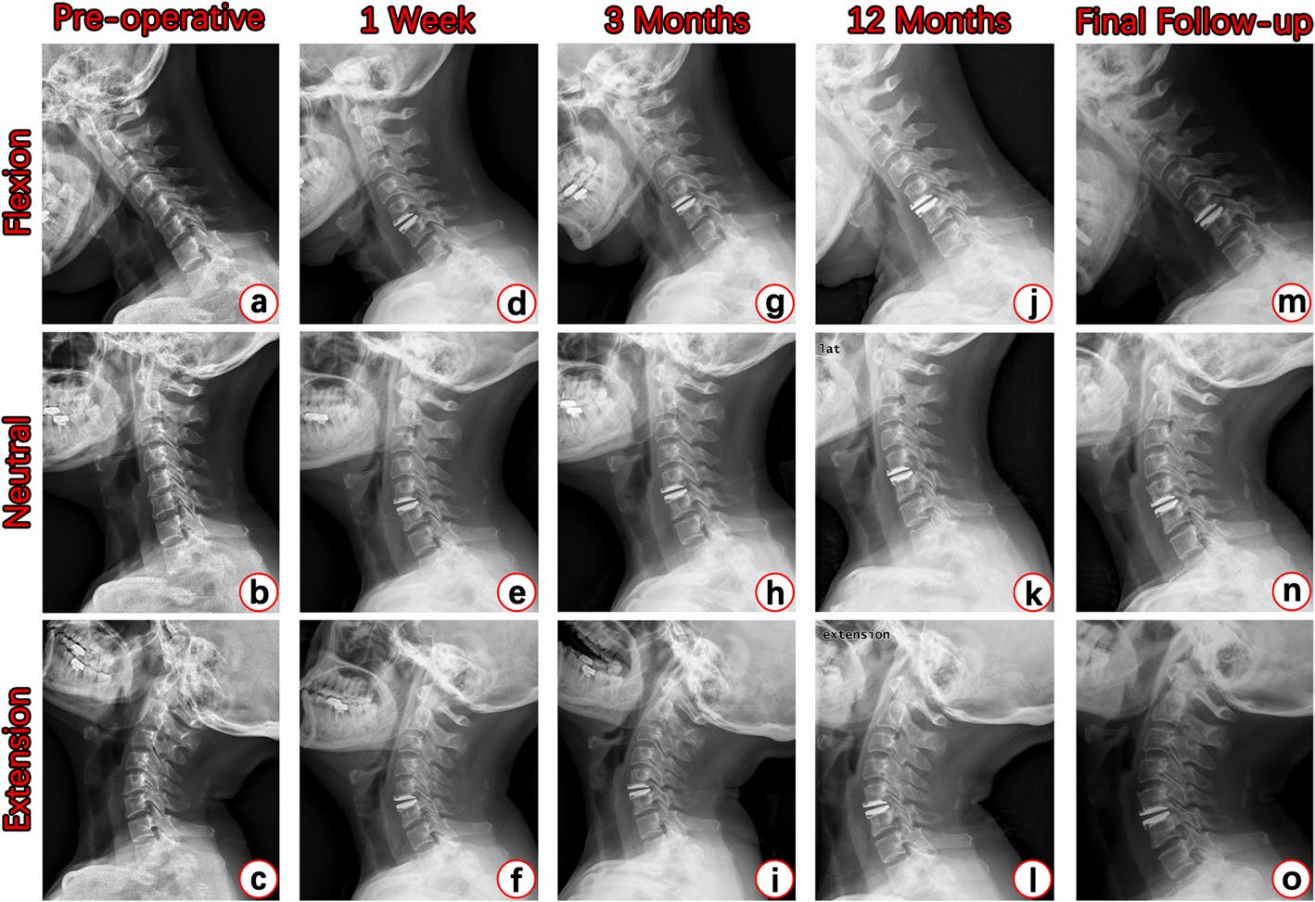
Radiological Results of Typical Case with Pretic-I. Figure 2 showed the radiological results of one typical case with Pretic-I pre-operatively and 1 week, 3 months, 12 months, 60 months post-operatively. **a**-**c** Cervical sagittal ROM condition at pre-operative stage. **d**-**f** Cervical sagittal ROM condition at 1 week post-operatively. **g**-**i** Cervical sagittal ROM condition at 3 months post-operatively. **j**-**l** Cervical sagittal ROM condition at 12 months post-operatively. **m**-**o** Cervical sagittal ROM condition at the final follow-up

As shown in Table 7, the Shell Angle of Pretic-I group decreased from 3.03° ± 3.68° to 2.23° ± 4.10° (*p* = 0.1988), while that of Discover group decreased from 0.75° ± 3.42° to 0.09° ± 4.44° (*p* = 0.1820), showing no significant difference. The C2–7 Cobb angle of the Discover group increased from 12.05° ± 12.17° pre-operatively to 19.32° ± 9.32° at the final follow-up (*p* = 0.0104, Statistical Power = 98.20%); while in the Pretic-I group, it increased from 15.08° ± 11.75° pre-operatively to 18.00° ± 10.61° at the final follow-up (*p* = 0.3079). C2–7 ROM and FSU ROM showed no significant change at the final follow-up in both groups (Table 7). With more detailed analysis, the ROM was divided into flexion ROM and extension ROM, which also showed no significant changes. Figure 2 shows a typical case of patient with Pretic-I, demonstrating that after CDA with Pretic-I, the cervical curvature of the patient was greatly maintained and even improved; the cervical total ROM and FSU ROM were well preserved along the whole follow-up period. Looking at the whole follow-up period, as shown in Table 8, there was no significant difference with valid statistical power for each parameter of the two groups.

**Table 7:** Radiological changes of the final follow-up

Items	Groups	Pre-operative	1 Week	Final Follow-up	***P*** Value	Statistical Power
**Shell Angle (°)**	Discover (*n* = 32)	(−)	0.75 ± 3.42	0.09 ± 4.44	0.1820	(−)
	Pretic-I (*n* = 32)	(−)	3.03 ± 3.68	2.23 ± 4.10	0.1988	(−)
**C2–7 Cobb Angle (°)**	Discover (*n* = 32)	12.05 ± 12.17	(−)	19.32 ± 9.32	0.0104*	98.20%
	Pretic-I (*n* = 32)	15.08 ± 11.75	(−)	18.00 ± 10.61	0.3079	(−)
**C2–7 ROM**						
Flexion ROM (°)	Discover (*n* = 32)	32.63 ± 8.86	(−)	31.49 ± 9.37	0.6258	(−)
	Pretic-I (*n* = 32)	34.01 ± 11.42	(−)	33.35 ± 8.77	0.7993	(−)
Extension ROM (°)	Discover (*n* = 32)	18.94 ± 8.19	(−)	19.13 ± 10.19	0.9366	(−)
	Pretic-I (*n* = 32)	18.47 ± 9.07	(−)	19.95 ± 8.60	0.5125	(−)
Total ROM (°)	Discover (*n* = 32)	51.57 ± 12.36	(−)	50.62 ± 10.39	0.7449	(−)
	Pretic-I (*n* = 32)	52.48 ± 14.31	(−)	53.30 ± 11.71	0.8062	(−)
**FSU ROM**						
Flexion ROM (°)	Discover (*n* = 32)	7.04 ± 4.02	(−)	6.18 ± 3.76	0.3856	(−)
	Pretic-I (*n* = 32)	8.35 ± 3.88	(−)	6.60 ± 4.14	0.0919	(−)
Extension ROM (°)	Discover (*n* = 32)	4.83 ± 3.91	(−)	4.19 ± 2.84	0.4580	(−)
	Pretic-I (*n* = 32)	3.85 ± 2.65	(−)	4.12 ± 2.93	0.7035	(−)
Total ROM (°)	Discover (*n* = 32)	11.07 ± 5.36	(−)	10.36 ± 4.79	0.5870	(−)
	Pretic-I (*n* = 32)	12.20 ± 4.52	(−)	10.73 ± 4.45	0.2002	(−)

*ROM* Range of motion, *FSU* Functional segment unit, *(−)* Without valid data

* indicates that the data has a *P* value less than 0.05

**Table 8:** Follow-up results of radiological parameters

Items	Groups	Pre-operative		1 Week		3 Months		12 Months		Final Follow-up	
**Shell Angle (°)**	Discover (*n* = 32)	(−)	(−)	0.75 ± 3.42	***p*** = 0.0141* **SP = 71.48%**	0.91 ± 3.80	*p* = 0.0811	0.51 ± 4.30	*p* = 0.0863	0.09 ± 4.44	*p* = 0.0529
	Pretic-I (*n* = 32)	(−)		3.03 ± 3.68		2.64 ± 3.85		2.35 ± 3.99		2.23 ± 4.10	
**C2–7 Cobb Angle (°)**	Discover (*n* = 32)	12.05 ± 12.17	*p* = 0.3223	16.47 ± 9.46	*p* = 0.5947	17.11 ± 10.26	*p* = 0.5805	16.39 ± 9.76	*p* = 0.8763	19.32 ± 9.32	*p* = 0.6043
	Pretic-I (*n* = 32)	15.08 ± 11.75		17.84 ± 10.62		15.47 ± 12.83		16.82 ± 11.83		18.00 ± 10.61	
**C2–7 ROM**											
Flexion ROM (°)	Discover (*n* = 32)	32.63 ± 8.86	*p* = 0.5973	23.44 ± 10.48	*p* = 0.9865	32.48 ± 8.17	*p* = 0.2451	32.29 ± 10.74	*p* = 0.7345	31.49 ± 9.37	*p* = 0.4245
	Pretic-I (*n* = 32)	34.01 ± 11.42		23.48 ± 10.97		29.41 ± 12.08		33.18 ± 9.71		33.35 ± 8.77	
Extension ROM (°)	Discover (*n* = 32)	18.94 ± 8.19	*p* = 0.8321	12.80 ± 7.06	*p* = 0.4442	17.01 ± 11.08	*p* = 0.4040	22.49 ± 11.61	*p* = 0.1712	19.13 ± 10.19	*p* = 0.7320
	Pretic-I (*n* = 32)	18.47 ± 9.07		11.55 ± 5.66		19.28 ± 10.21		18.47 ± 11.25		19.95 ± 8.60	
Total ROM (°)	Discover (*n* = 32)	51.57 ± 12.36	*p* = 0.7892	36.24 ± 12.67	*p* = 0.6772	49.49 ± 12.65	*p* = 0.8173	54.78 ± 13.71	*p* = 0.3593	50.62 ± 10.39	*p* = 0.3447
	Pretic-I (*n* = 32)	52.48 ± 14.31		35.03 ± 9.84		48.69 ± 14.44		51.64 ± 13.00		53.30 ± 11.71	
**FSU ROM**											
Flexion ROM (°)	Discover (*n* = 32)	7.04 ± 4.02	*p* = 0.1964	5.60 ± 3.74	*p* = 0.8755	6.97 ± 3.52	*p* = 0.3079	7.11 ± 3.24	*p* = 0.5085	6.18 ± 3.76	*p* = 0.6706
	Pretic-I (*n* = 32)	8.35 ± 3.88		5.45 ± 3.61		6.09 ± 3.25		6.55 ± 3.29		6.60 ± 4.14	
Extension ROM (°)	Discover (*n* = 32)	4.83 ± 3.91	*p* = 0.2512	4.32 ± 2.90	*p* = 0.3818	4.51 ± 3.98	*p* = 0.7049	4.68 ± 4.03	*p* = 0.7688	4.19 ± 2.84	*p* = 0.9319
	Pretic-I (*n* = 32)	3.85 ± 2.65		3.70 ± 2.64		4.24 ± 2.84		4.40 ± 3.30		4.12 ± 2.93	
Total ROM (°)	Discover (*n* = 32)	11.07 ± 5.36	*p* = 0.3702	9.92 ± 4.62	*p* = 0.4755	11.48 ± 4.41	*p* = 0.2711	11.78 ± 5.51	*p* = 0.5389	10.36 ± 4.79	*p* = 0.7560
	Pretic-I (*n* = 32)	12.20 ± 4.52		9.15 ± 3.76		10.33 ± 3.70		10.95 ± 5.03		10.73 ± 4.45	

*ROM* Range of motion, *FSU* Functional segment unit, *SP* Statistical power, *(−)* Without valid data

* indicates that the data has a *P* value less than 0.05

### Complications

As shown in Table 9, the flexion ROM, extension ROM and total ROM in the adjacent segment of the two groups were very close at each follow-up point. Furthermore, as shown in Table 10, at the final follow-up, ROM in the upper and lower FSU of both groups did not significantly change compared with that pre-operative data. At each follow-up point, as shown in Table 11, there were no statistical differences between two groups in the listed items of ASD characteristics. In addition, there was similar incidence of CDDD-related symptoms in the adjacent segments between the two groups. Therefore, the incidence of cases that met at least one of the above descriptions was calculated, showing that at the final follow-up, the total incidence of ASD (ASDeg and ASDis) in upper segment was 21.88% (7/32) in the Discover group and 18.75% (6/32) in the Pretic-I group, with no significant difference (*p* = 0.7560). The total incidence of lower segment ASD (ASDeg and ASDis) was 15.63% (5/32) in the Discover group and 18.75% (6/32) in the Pretic-I group, also with no significant difference (*p* = 0.7404). The incidence of HO between the two groups was further compared. As shown in Table 12, at the final follow-up, the HO incidence was 65.63% (21/32) in the Discover group and 46.88% (15/32) in the Pretic-I group, respectively. There was no significant difference between the two groups (*p* = 0.1306). The incidence of Grade III-IV HO was 12.50% (4/32) in the Pretic-I group, which is significantly lower than the 34.38% (11/32) of Discover group (*p* = 0.0389, Statistical Power = 95.36%). The other related clinical complications of CDA between the two groups were further analyzed and compared. Although the incidence of subsidence of the final follow-up in the Pretic-I group (12.50%, 4/32) appears to be lower than that in the Discover group (3.13%, 1/32), there is no significant difference (*p* = 0.1623). As shown in Table 12, a small number of patients in both groups had suffered from dysphagia 1 week after surgery (12.50% in Discover group, 9.38% in Pretic-I group, *p* = 0.6888). Fortunately, all symptoms of dysphagia gradually disappeared after 3 months without specific treatment.

**Table 9:** Range of motion of adjacent segments

Items	Groups	Pre-operative		1 Week		3 Months		12 Months		Final Follow-up	
**FSU ROM of Upper Adjacent Segment**											
Flexion ROM (°)	Discover (*n* = 32)	6.20 ± 4.24	*p* = 0.5677	6.12 ± 4.11	*p* = 0.0995	8.30 ± 4.53	*p* = 0.0773	6.86 ± 3.56	*p* = 0.7426	7.26 ± 4.06	*p* = 0.8000
	Pretic-I (*n* = 32)	6.79 ± 3.87		4.49 ± 3.51		6.50 ± 3.26		7.16 ± 3.63		7.54 ± 4.38	
Extension ROM (°)	Discover (*n* = 32)	6.24 ± 4.71	*p* = 0.2225	4.24 ± 2.83	*p* = 0.2645	4.47 ± 3.59	*p* = 0.3364	5.70 ± 3.70	*p* = 0.4936	4.81 ± 3.63	*p* = 0.8942
	Pretic-I (*n* = 32)	4.99 ± 3.05		3.53 ± 2.05		5.32 ± 3.34		5.08 ± 3.33		4.92 ± 3.12	
Total ROM (°)	Discover (*n* = 32)	12.43 ± 6.10	*p* = 0.6463	10.35 ± 4.69	***p*** = 0.0283* **SP = 61.27%**	12.77 ± 5.26	*p* = 0.4475	12.56 ± 5.69	*p* = 0.8134	12.07 ± 5.29	*p* = 0.7703
	Pretic-I (*n* = 32)	11.78 ± 4.92		8.02 ± 3.37		11.82 ± 4.44		12.24 ± 4.71		12.46 ± 3.12	
**FSU ROM of Lower Adjacent Segment**											
Flexion ROM (°)	Discover (*n* = 32)	7.68 ± 3.41	*p* = 0.7073	5.30 ± 2.83	*p* = 0.7325	6.83 ± 3.14	*p* = 0.9586	7.46 ± 2.84	*p* = 0.2210	7.02 ± 3.64	*p* = 0.2734
	Pretic-I (*n* = 32)	8.14 ± 5.82		5.53 ± 2.32		6.79 ± 3.28		6.45 ± 3.54		6.15 ± 2.42	
Extension ROM (°)	Discover (*n* = 32)	4.19 ± 3.59	*p* = 0.3486	2.81 ± 2.06	*p* = 0.5358	3.98 ± 3.18	*p* = 0.3345	3.99 ± 3.38	*p* = 0.8579	3.75 ± 2.76	*p* = 0.5008
	Pretic-I (*n* = 32)	3.46 ± 2.41		3.13 ± 1.98		3.32 ± 2.06		4.12 ± 2.33		4.21 ± 2.61	
Total ROM (°)	Discover (*n* = 32)	11.87 ± 4.92	*p* = 0.8473	8.11 ± 3.12	*p* = 0.4517	10.81 ± 3.77	*p* = 0.4610	11.45 ± 4.45	*p* = 0.4357	10.77 ± 4.22	*p* = 0.6916
	Pretic-I (*n* = 32)	11.59 ± 6.06		8.65 ± 2.51		10.11 ± 3.71		10.58 ± 4.32		10.37 ± 3.75	

*ROM* Range of motion, *FSU* Functional segment unit, *SP* Statistical power

* indicates that the data has a *P* value less than 0.05

**Table 10:** Changes in range of motion of adjacent segments of the final follow-up

Items	Groups	Pre-operative	Final Follow-up	***P*** Value	Statistical Power
**FSU ROM of Upper Adjacent Segment**					
Flexion ROM (°)	Discover (*n* = 32)	6.20 ± 4.24	7.26 ± 4.06	0.2693	(−)
	Pretic-I (*n* = 32)	6.79 ± 3.87	7.54 ± 4.38	0.4141	(−)
Extension ROM (°)	Discover (*n* = 32)	6.24 ± 4.71	4.81 ± 3.63	0.0614	(−)
	Pretic-I (*n* = 32)	4.99 ± 3.05	4.92 ± 3.12	0.9225	(−)
Total ROM (°)	Discover (*n* = 32)	12.43 ± 6.10	12.07 ± 5.29	0.7524	(−)
	Pretic-I (*n* = 32)	11.78 ± 4.92	12.46 ± 3.12	0.4892	(−)
**FSU ROM of Lower Adjacent Segment**					
Flexion ROM (°)	Discover (*n* = 32)	7.68 ± 3.41	7.02 ± 3.64	0.3737	(−)
	Pretic-I (*n* = 32)	8.14 ± 5.82	6.15 ± 2.42	0.0449*	75.50%
Extension ROM (°)	Discover (*n* = 32)	4.19 ± 3.59	3.75 ± 2.76	0.4143	(−)
	Pretic-I (*n* = 32)	3.46 ± 2.41	4.21 ± 2.61	0.1973	(−)
Total ROM (°)	Discover (*n* = 32)	11.87 ± 4.92	10.77 ± 4.22	0.1007	(−)
	Pretic-I (*n* = 32)	11.59 ± 6.06	10.37 ± 3.75	0.1705	(−)

*ROM* Range of motion, *FSU* Functional segment unit, *(−)* Without valid data

* indicates that the data has a *P* value less than 0.05

**Table 11:** Evaluation of adjacent segment disease

Items	Groups	Pre-operative		1 Week		3 Months		12 Months		Final Follow-up	
**Incidence of ASDeg**											
Intervertebral Space Narrowing>25%											
Upper Segment	Discover (*n* = 32)	(−)	(−)	0.00%(0/32)	(−)	0.00%(0/32)	(−)	3.13%(1/32)	*p* = 0.3135	6.25%(2/32)	*p* = 0.3911
	Pretic-I (*n* = 32)	(−)		0.00%(0/32)		0.00%(0/32)		0.00%(0/32)		12.50%(4/32)	
Lower Segment	Discover (*n* = 32)	(−)	(−)	0.00%(0/32)	(−)	0.00%(0/32)	(−)	0.00%(0/32)	(−)	12.50%(4/32)	*p* = 1.0000
	Pretic-I (*n* = 32)	(−)		0.00%(0/32)		0.00%(0/32)		0.00%(0/32)		12.50%(4/32)	
New/Enlarged Osteophytes											
Upper Segment	Discover (*n* = 32)	(−)	(−)	0.00%(0/32)	(−)	0.00%(0/32)	(−)	6.25%(2/32)	*p* = 0.1508	18.75%(6/32)	*p* = 0.4911
	Pretic-I (*n* = 32)	(−)		0.00%(0/32)		0.00%(0/32)		0.00%(0/32)		12.50%(4/32)	
Lower Segment	Discover (*n* = 32)	(−)	(−)	0.00%(0/32)	(−)	0.00%(0/32)	(−)	0.00%(0/32)	*p* = 0.3135	12.50%(4/32)	*p* = 0.7192
	Pretic-I (*n* = 32)	(−)		0.00%(0/32)		0.00%(0/32)		3.13%(1/32)		15.63%(5/32)	
New Disc Herniation											
Upper Segment	Discover (*n* = 32)	(−)	(−)	0.00%(0/32)	(−)	0.00%(0/32)	(−)	9.38%(3/32)	*p* = 0.6414	18.75%(6/32)	*p* = 0.7404
	Pretic-I (*n* = 32)	(−)		0.00%(0/32)		0.00%(0/32)		6.25%(2/32)		12.50%(5/32)	
Lower Segment	Discover (*n* = 32)	(−)	(−)	0.00%(0/32)	(−)	0.00%(0/32)	(−)	3.13%(1/32)	*p* = 1.0000	9.38%(3/32)	*p* = 1.0000
	Pretic-I (*n* = 32)	(−)		0.00%(0/32)		0.00%(0/32)		3.13%(1/32)		9.38%(3/32)	
Endplate Sclerosis											
Upper Segment	Discover (*n* = 32)	(−)	(−)	0.00%(0/32)	(−)	0.00%(0/32)	(−)	3.13%(1/32)	p = 1.0000	3.13%(1/32)	*p* = 0.3017
	Pretic-I (*n* = 32)	(−)		0.00%(0/32)		0.00%(0/32)		3.13%(1/32)		9.38%(3/32)	
Lower Segment	Discover (*n* = 32)	(−)	(−)	0.00%(0/32)	(−)	0.00%(0/32)	(−)	0.00%(0/32)	*p* = 0.3135	6.25%(2/32)	*p* = 0.5543
	Pretic-I (*n* = 32)	(−)		0.00%(0/32)		0.00%(0/32)		3.13%(1/32)		3.13%(1/32)	
Anterior/Posterior Longitudinal Ligament Calcification											
Upper Segment	Discover (*n* = 32)	(−)	(−)	0.00%(0/32)	(−)	0.00%(0/32)	(−)	3.13%(1/32)	*p* = 1.0000	6.25%(2/32)	*p* = 0.5543
	Pretic-I (*n* = 32)	(−)		0.00%(0/32)		0.00%(0/32)		3.13%(1/32)		3.13%(1/32)	
Lower Segment	Discover (*n* = 32)	(−)	(−)	0.00%(0/32)	(−)	0.00%(0/32)	(−)	6.25%(2/32)	*p* = 0.5543	9.38%(3/32)	*p* = 0.6414
	Pretic-I (*n* = 32)	(−)		0.00%(0/32)		0.00%(0/32)		3.13%(1/32)		6.25%(2/32)	
**Incidence of ASDis**											
Upper Segment	Discover (*n* = 32)	6.25%(2/32)	*p* = 0.5543	6.25%(2/32)	*p* = 0.5543	6.25%(2/32)	*p* = 0.5543	6.25%(2/32)	*p* = 0.5543	15.63%(5/32)	*p* = 0.7192
	Pretic-I (*n* = 32)	3.13%(1/32)		3.13%(1/32)		3.13%(1/32)		3.13%(1/32)		12.50%(4/32)	
Lower Segment	Discover (*n* = 32)	3.13%(1/32)	*p* = 1.0000	3.13%(1/32)	*p* = 1.0000	3.13%(1/32)	*p* = 1.0000	3.13%(1/32)	*p* = 1.0000	6.25%(2/32)	*p* = 1.0000
	Pretic-I (*n* = 32)	3.13%(1/32)		3.13%(1/32)		3.13%(1/32)		3.13%(1/32)		6.25%(2/32)	
**Total Incidence of ASD**											
Upper Segment	Discover (*n* = 32)	6.25%(2/32)	*p* = 0.5543	6.25%(2/32)	*p* = 0.5543	6.25%(2/32)	*p* = 0.5543	15.63%(5/32)	*p* = 0.4479	21.88%(7/32)	*p* = 0.7560
	Pretic-I (*n* = 32)	3.13%(1/32)		3.13%(1/32)		3.13%(1/32)		9.38%(3/32)		18.75%(6/32)	
Lower Segment	Discover (*n* = 32)	3.13%(1/32)	*p* = 1.0000	3.13%(1/32)	*p* = 1.0000	3.13%(1/32)	*p* = 1.0000	9.38%(3/32)	*p* = 1.0000	15.63%(5/32)	*p* = 0.7404
	Pretic-I (*n* = 32)	3.13%(1/32)		3.13%(1/32)		3.13%(1/32)		9.38%(3/32)		18.75%(6/32)	

*ASD* Adjacent segment disease, *(−)* Without valid data

**Table 12:** Incidence of complications

Items	Groups	Pre-operative		1 Week		3 Months		12 Months		Final Follow-up	
**HO**											
Grade I	Discover (*n* = 32)	0.00%(0/32)	(−)	0.00%(0/32)	(−)	0.00%(0/32)	(−)	9.38%(3/32)	*p* = 0.6414	15.63%(5/32)	*p* = 1.0000
	Pretic-I (*n* = 32)	0.00%(0/32)		0.00%(0/32)		0.00%(0/32)		6.25%(2/32)		15.63%(5/32)	
Grade II	Discover (*n* = 32)	0.00%(0/32)	(−)	0.00%(0/32)	(−)	0.00%(0/32)	(−)	6.25%(2/32)	*p* = 1.0000	15.63%(5/32)	*p* = 0.7404
	Pretic-I (*n* = 32)	0.00%(0/32)		0.00%(0/32)		0.00%(0/32)		6.25%(2/32)		18.75%(6/32)	
Grade III	Discover (*n* = 32)	0.00%(0/32)	(−)	0.00%(0/32)	(−)	0.00%(0/32)	(−)	18.75%(6/32)	*p* = 0.2807	21.88%(7/32)	*p* = 0.0722
	Pretic-I (*n* = 32)	0.00%(0/32)		0.00%(0/32)		0.00%(0/32)		9.38%(3/32)		6.25%(2/32)	
Grade IV	Discover (*n* = 32)	0.00%(0/32)	(−)	0.00%(0/32)	(−)	0.00%(0/32)	(−)	0.00%(0/32)	(−)	12.50%(4/32)	*p* = 0.3911
	Pretic-I (*n* = 32)	0.00%(0/32)		0.00%(0/32)		0.00%(0/32)		0.00%(0/32)		6.25%(2/32)	
Grade III + IV	Discover (*n* = 32)	0.00%(0/32)	(−)	0.00%(0/32)	(−)	0.00%(0/32)	(−)	18.75%(6/32)	*p* = 0.2807	34.38%(11/32)	***p*** = 0.0389* **SP = 95.36%**
	Pretic-I (*n* = 32)	0.00%(0/32)		0.00%(0/32)		0.00%(0/32)		9.38%(3/32)		12.50%(4/32)	
Total	Discover (*n* = 32)	0.00%(0/32)	(−)	0.00%(0/32)	(−)	0.00%(0/32)	(−)	34.38%(11/32)	*p* = 0.2661	65.63%(21/32)	*p* = 0.1306
	Pretic-I (*n* = 32)	0.00%(0/32)		0.00%(0/32)		0.00%(0/32)		21.88%(7/32)		46.88%(15/32)	
**Subsidence**	Discover (*n* = 32)	(−)	(−)	0.00%(0/32)	(−)	0.00%(0/32)	(−)	9.38%(3/32)	*p* = 0.3107	12.50%(4/32)	*p* = 0.1623
	Pretic-I (*n* = 32)	(−)		0.00%(0/32)		0.00%(0/32)		3.13%(1/32)		3.13%(1/32)	
**Displacement**	Discover (*n* = 32)	(−)	(−)	0.00%(0/32)	(−)	0.00%(0/32)	(−)	0.00%(0/32)	(−)	6.25%(2/32)	*p* = 0.5543
	Pretic-I (*n* = 32)	(−)		0.00%(0/32)		0.00%(0/32)		0.00%(0/32)		3.13%(1/32)	
**Dysphagia**	Discover (*n* = 32)	0.00%(0/32)	(−)	12.50%(4/32)	*p* = 0.6888	3.13%(1/32)	*p* = 1.0000	0.00%(0/32)	(−)	0.00%(0/32)	(−)
	Pretic-I (*n* = 32)	0.00%(0/32)		9.38%(3/32)		3.13%(1/32)		0.00%(0/32)		0.00%(0/32)	

*HO* Heterotopic ossification, *(−)* Without valid data

* indicates that the data has a *P* value less than 0.05

## Discussion

CDA has become one of the surgical options for the treatment of CDDD, and is even superior to ACDF in some cases. However, it is possible for artificial cervical discs to fail, mainly due to prosthesis displacement, subsidence, and heterotopic ossification. Martin et al. [9] proposed the concept of footprint mismatch in 2012, that is, if the end plate of the artificial cervical disc prosthesis does not fit well with the bone surface of the corresponding vertebral body, the related complication incidence will be significantly increased. Therefore, how to better match the two has become a point that needs to be paid attention to in the development of artificial cervical disc prosthesis.

Our center has modified and redesigned the existing cervical disc prosthesis, known as Pretic-I in this article, and conducted a 5-year follow-up and comparative study among a limited number of patients. The evaluation focused on the following aspects: the difficulty of implantation, the clinical efficacy, the ability to maintain cervical curvature and ROM, and the incidence of related complications.

In the first two parts, there was no significant difference in peri-operative indicators between the Pretic-I and Discover groups, indicating that the difficulty of operation was similar, and the recovery rate of patients receiving the two types of prostheses was also show no differences. At the same time, CDA with two prostheses were both associated with great clinical outcomes, and there was no significant difference in follow-up between the two groups at the same time point, assuring the clinical efficacy of Pretic-I prosthesis.

The radiological outcomes of the two groups were compared as an important part. By comparing the results of pre-operative and final follow-up, it was found that both Discover and Pretic-I could improve cervical curvature. In addition, Shell angles of patients in both groups did not change significantly from 0.75° ± 3.42° to 0.09° ± 4.44° in Discover group and from 3.03° ± 3.68° to 2.23° ± 4.10° in Pretic-I group from 1 week after surgery to the final follow-up. Those results suggested that both prostheses can improve cervical curvature to a certain extent, and the prostheses themselves can maintain a perfect opening status. Similarly, by comparing the pre-operative and final follow-up results, it was found that after 5 years of observation, the C2–7 ROM of the both groups changed slightly along the follow-up. It was observed that the C2–7 ROM and FSU ROM of the two groups maintained the same size as before surgery, and there was no significant difference between the two groups at each follow-up point, indicating that both prostheses can maintain the original ROM of cervical spine, which also best reflects the value of CDA surgery.

Finally, the CDA-related complications of the two groups were analyzed and compared. The changes in adjacent FSU ROM were firstly compared, revealing that all of the data had no significant difference with valid statistical power at each follow-up point. Accordingly, it was believed that the ROM of adjacent segments for the two groups did not change significantly during the whole follow-up, indicating that there was no obvious compensatory increase in adjacent segment ROM. Next, the degenerative radiological features and CDDD-related symptoms in adjacent segments of the two groups were analyzed to evaluate the incidences of ASDeg and ASDis, respectively, also showing no significant differences, which indicated that ASD incidences were similar between both groups. The incidence of high-grade HO in Pretic-I was significantly lower than in the Discover group (12.50% vs. 34.38%, *p* = 0.0389, Statistical Power = 95.36%), as was the incidence of prosthetic subsidence, although there was no statistically significant difference (3.13% vs. 12.50%, *p* = 0.1306). This may reflect, to some extent, that the prosthetic endplate of Pretic-I is better matched with the bone surface of the vertebral body. No more differences in incidences of other complications were found.

However, there are still many limitations to this study. First of all, although we have fully balanced the basic data of all the included patients, there is still some bias due to the lack of “randomization”. After confirming the clinical safety and efficacy of Pretic-I prosthesis in this study, we will adopt the method of “randomized controlled study” in the follow-up large-scale clinical trial for further verification. Secondly, limited by the scale of clinical application, the number of patients with 5-year follow-up data is small and needs to be further supplemented.

In this study, it was the first time to report the clinical efficacy of Pretic-I with a long-term follow-up. From the results obtained so far, it can be seen that Pretic-I has a good clinical efficacy and, after CDA with Pretic-I, the ROM of the cervical spine and FSU can be well maintained. At the same time, the incidence of post-operative complications in the Pretic-I group was also acceptable, and even showed some advantages in the high-grade HO. Generally, we believe that Pretic-I could be one safe and effective alternative to cervical disc prosthesis in the future.

## Supplementary Information

**Additional file 1.**

## Data Availability

The original data are available and attached to the submission as the supplement.

## Abbreviations

ACDF: Anterior cervical discectomy and fusion
ASDeg: Adjacent segment degeneration
ASDis: Adjacent segment disease
CDA: Cervical disc arthroplasty
CDDD: Cervical disc degenerative disease
CT: Computed tomography
FSU: Functional segment unit
HO: Heterotopic ossification
JOA: Japanese Orthopaedic Association Score
NDI: Neck disability index
ROM: Range of motion
VAS: Visual analog score
